# Therapeutics

**Published:** 1824-09-01

**Authors:** 


### Therapœia.

Pharmaca nulla valent, nisi quæ sint commoda causis.

1. *Hydrocephalus Internus.* In the last Number of the Edinburgh Medical Journal there is a short, but in our minds, an important communication on the subject of hydrocephalus *internus*, or as we would term it, hydrocephalus *acutus*, from the pen of Dr. Maxwell of Dumfries. Previously to our author's entrance on private practice he saw about twenty-five cases of this disease all terminating fatally. He naturally concluded therefore that the *methodus medendi* was inefficient, and that some new or more active mode of treatment must be resorted to, if success were to be looked for. In many of these fatal cases the little patients had been bled with leeches and from the arm—purged, &c. It was therefore determined to abstract blood in a more bold and decided manner than had yet been tried. Out of about ninety cases treated in the manner to be described, sixty recovered—a proportion greater certainly than is usually observed in ordinary practice.

Dr. Maxwell has selected only two cases out of nearly ninety, in order not only to shew that the disease was really hydrocephalus acutus, as far as symptoms can prove, but also to illustrare the mode of practice pursued. One of these two cases will be sufficient for our purpose.

"Master J., æt. 7, a healthy boy, became dull and indisposed to his usual exercise, complaining of headach, which, with much languor, increased during six or eight days. The bowels were frequently moved by purgatives. At this period pain in the head became more distressing, and the bowels were difficultly moved. The boy showed no inclination to leave the house:—he rested frequently his head in his hands upon a table, or in a kneeling posture on a chair; the muscular power of his limbs began to fail; the pulse became rapid; pain of the head excessive, with occasional remissions, and during these, constant drowsiness came on. Still, however, there was a considerable disposition to take food, although it was passed half digested. The mind now became indistinct, with inability to articulate; vision was imperfect; the evacuations took place without his attention; squinting next appeared; pulse 160—frequent expression of pain in the head; muscles in the neck became supple, the head rolling upon the breast and shoulders, with immobility of the pupils and total want of expression in the eyes; the face pale and inanimate. Mr. J., his father, a medical gentleman, requested that I would take the sole direction of the case, observing, that he was well aware that extreme bleeding was the only means by which the child could be recovered; desiring that it might be carried to whatever extent it might be thought necessary, and that, if the child should sink under such active treatment, he would still retain a warm sense of gratitude for the painful task which he had imposed on me. The time was fixed for the operation, and, with the father's consent, I invited my medical friends Dr. G., Mr. S., Mr. M. and Mr. St., to be present. The father, after providing every thing necessary, withdrew to a remote part of the house, waiting, with the anxiety of an affectionate parent, the fate of an only son.

"The boy was laid on a mattress, his head somewhat lower than the rest of the person, medical gentlemen holding each wrist; the jugular vein on the right side was opened; it bled rapidly—the stream was frequently interrupted to prevent fainting. The bleeding was continued till syncope began to take place: a little negus was then given; when the pulse revived the finger was removed from the orifice and the blood allowed to flow till the gentlemen agreed that the pulse could no longer be felt. The patient at this time had no appearance of life, and continued without the least symptom of animation for ten minutes, when he began to revive gradually: and in the evening more favourable symptoms appeared. His mind was remarkably improved, as well as his physical powers, being now able to articulate, although indistinctly, and to tell the hour on a watch. He had a tolerable night's rest, having taken frequently a little water-gruel and beef-tea. During the following day there was but little improvement. On the third day the bleeding was repeated in the left jugular vein, and a complete recovery followed." 13.

We think this document is highly deserving the attention of our brethren. Before water is actually effused—at least to any extent—bleeding must be the paramount measure, whether in young or in old.

---

2. *Prevention of Drunkenness.* Our readers are aware that the volatile alkali has been considered as a *remedy* for intoxication. We said, in a former number, that should this be the case, it is doubtful whether the antidote would not encourage the vice rather than tend to suppress it. In one of the foreign Journals it is stated that a German Physician (M. Brulh-Cramer) has discovered that the exhibition of diluted sulphuric acid, with occasional bitters, causes, at length, such a disgust towards brandy and other spirituous potations, as to eradicate the disposition to inebriety. If this should prove true, it would be a far more valuable discovery than that of a medicine which rendered a drunken man sober, and enabled him to return to his favourite potations with impunity.

3. *Cholera of India.* This terrible epidemic is spreading westward. It is already on the very confines of Europe, and will probably visit us before it ceases its ravages. Mr. Cormick writes from Tabriz, in Persia, under date of October 1822, at which time the cholera had got to the Western boundary of Persia, and was steadily advancing in the same direction. This gentleman confirms the general observation that, "there is not a vomiting and purging of bile, but of a whitish water, without taste or smell, resembling that in which rice had been boiled." Until alvine evacuations, of "a dark colour or bilious nature," were procured, no cure could be anticipated—"and this object gained, two thirds of the difficulty and danger of the disease are removed." This was discovered and proved many years before the epidemic broke out, by observations on sporadic cases in India.

"Calomel, sometimes alone, sometimes combined with opium, and opium alone, had this effect. Occasionally, when all these failed, injections, twice or thrice repeated, of laudanum and warm water, or rice water, one drachm in a pint, succeeded. I always gave calomel the first trial, as it possessed the additional advantage of bringing on a healthy action in the liver, (which was gorged with blood) and a secretion of bile, more readily and more effectually than any other medicine with which I am acquainted. As soon as the state of the stomach admitted of it, I gave six to ten grains of calomel, with ten grains of compound extract of colocynth, every hour, administering at the same time a stimulating injection, generally of salt and water. This medicine I used as, being small in quantity, it was less likely to bring on a recurrence of the vomiting. After three or four repetitions of it, if copious evacuations were not produced, which was seldom the case, I gave an ounce of castor oil, with as much peppermint water, every hour till this object was attained." 361.

Bleeding could not be attempted before some degree of reaction came on. It was then "of infinite use in relieving the head, removing the disposition to coma, and facilitating the return of the healthy secretions of the hepatic system." The same observations apply to warm bathing as to blood-letting. Latterly Mr. C. was led by experience "to place most confidence in pieces of blanket moistened in water, almost boiling, and constantly rubbed and tied about the legs and arms." Mr. C. ridicules the idea of the disease being contagious. He saw not an atom of reason for such an opinion. Speaking of Tabriz, Mr. Cormick observes,

"The atmosphere is generally clear, cold, and healthy; and if, in such a climate, this epidemic commits such ravages as almost to equal its effects in many parts of India, I much fear it will extend to Europe, where the crowded cities and great population will make it more severely felt than it has been in the scattered cities and scanty population of Persia." 365.

We should not be surprised if Mr. Cormick's apprehensions be one day verified.—*Med. Chir. Trans.* v. xii.

4. *Purpura Hæmorrhagica.* Dr. Whitlock Nichol has related another case of this kind "successfully treated with the oleum terebinthinæ." The patient was a young country girl, nine years of age, who had been in a delicate state for several months. On the 3d of March, petechiæ appeared on the surface of the body generally—on the 5th, the gums began to bleed. 7th. The urine became bloody. 8th. Discharge of blood from the bowels. 9th. Our author visited her, and found her in a state of great languor—the skin studded with purple spots, intermixed on the legs, with oval patches of ecchymoses. The urine and stools were full of blood, and this fluid was constantly oozing from the gums. Pulse 130 to 140. A six ounce mixture, containing an ounce of oil of turpentine, was ordered to be taken in doses of one table-spoonful every hour until some mitigation of the general hæmorrhagic disposition, after which the dose was to be given only every two hours. A turpentine glyster was also directed to be thrown up. Next day the symptoms were ameliorated. In two or three days the blood disappeared from the secretions and excretions, but the gums did not entirely heal for near a fortnight.

Dr. Nichol proposes that Captain Parry may be furnished with a cask of oil of turpentine to cure sea scurvy in the Arctic Regions. If Dr. Nichol had ever witnessed sea scurvy, its causes, progress, symptoms, and termination, he never would have made such a proposal.—*Med. Repos. No.* 6.

---

5. *Pharmaceutical Agents.* The impropriety of using any but the proper officinal names for pharmaceutical preparations, will be illustrated by the following ludicrous mistake. Dr. Vavaseur, in translating Mr. Cæsar Hawkins' paper on Syphilitic Ulcers of the Larynx, into the ARCHIVES GENERALES, comes to a part where Mr. Hawkins details a most dangerous and formidable case saved by large quantities of the "*Lisbon diet drink.*" This is a puzzler to Dr. Vavasseur—and after racking his brains, he translates it—"*shin of beef soup,*" adding the following learned note at the bottom of the page.—

"Ce qu'on nomme en Anglais *diet drink*, est une *decoction très legere de viande de bœuf;* mais j'ignore quelle est la modification particuliere dont il s'agit, indiquée par le mot *Lisbon.*"

This treatment of syphilitic ulcers, in England, by "*skin-of-beef-soup*" will now make the tour of Europe at least, and excite no small merriment and surprise among the wits of the continental profession, who will never dream that Mr. Hawkins meant the compound decoction of sarsaparilla.

---

6. *Acute Rheumatism.* M. Gosse of Geneva, has published a case of acute rheumatism treated by calomel and opium to ptyalism, with a couple of bleedings. The rheumatism ceased as soon as salivation was established. There is nothing new in this. But it appears M. Gosse has made some chemical experiments on the blood of persons under the influence of mercury, and found such blood contained much less of albumen than other blood—that there was less cruor—that it was more liquid—and, in short, less inflammatory.

Dr. Farre, Mr. Travers, and many others, have long entertained similar opinions; but we are not aware that chemical experiments have been made on the blood of persons who had taken mercury in this country. The subject is worth pursuing, and is not difficult to investigate.

---

7. *Tartrite of Antimony in large Doses.* In our last Periscope, we gave the experience of Dr. Fontaneilles on the practice of Rassori, in acute diseases. We have now (Archives Generales, Avril) the testimony of the celebrated Laennec, as published by his pupil Dr. Delagarde. Several cases are related by the latter, as treated in the Clinique of La Charité. But Laennec does not trust entirely to antimony in pneumonia. He prescribes moderate blood-letting. And whereas Rassori commenced with doses of 24 grains of the tartrite of antimony, in the twenty-four hours, Laennec begins with four or six grains dissolved in four or six half-glassfuls of orange flower water well sweetened, in the day and night—gradually augmenting the dose according to the "tolerance" evinced by the patient. In exhibiting this medicine, indeed, we have no other guide but experience in the individual case.

Of the above solution Laennec exhibits half a glassful every two hours. Frequently the first dose determines evacuations, upwards or downwards—and frequently the second dose checks these evacuations—if not, the third or fourth. We need not despair of seeing the "tolerance" established, unless excessive evacuations continue longer than this period, when, of course, we should desist. On the second day, (if the medicine be well borne the first) the quantity of the antimony is augmented, or even doubled. Where it is not well borne, M. Laennec is in the habit of combining some syrup of the white poppy with the menstruum. It is very rare, says Dr. Delagarde, that complete tolerance is not established by the second or third day, after which, the doses of the medicine may be carried to a very great height, without any inconvenience—till a certain period, of which no prediction can be formed, when the smallest quantity of the medicine cannot be borne. It is then to be entirely left off.

The cases published under Dr. Laennec's authority, and which are sufficiently numerous, of pulmonic inflammation treated principally by antimony, but assisted occasionally by venesection, do not impress us very favourably with the plan pursued. They were much more tedious than cases of the same description usually are in this country—and this, we think, is a very serious objection, considering how desirable it is to clear the chest as soon as possible of inflammation, when it exists, in order to prevent any change of structure in the lungs or their investing membranes which may afterwards predispose to the same or to other more formidable diseases. M. Laennec, it appears, has employed the same plan, not only in acute pneumonia, but in apoplexy, articular rheumatism, acute hydrocephalus, chorea, and other maladies. To this mode of treatment in any of the above diseases, excepting rheumatism and chorea, we have to urge the same objections, *a fortiori;* because the *cito* is almost indispensible for the *tute* in such cases. At the same time we think that the verification of Rassori's plans in other parts of the continent besides Italy, ought to prove a sufficient inducement to practitioners in this country (in hospital practice—for we think it generally inapplicable to private practice, on account of the watchful attention required in each case) to avail themselves of a more extended use of antimony as an auxiliary in acute inflammations. It is with this view we have laid the above observations before our readers, as an appendix to the article at page 221 of this volume.

---

8. *Emetine in Violets.* As the bee extracts honey from the most poisonous plants, so vegetable chemistry promises to extract poison from the most mellifluous. In this respect, " the poisonous henbane and the fragrant rose" will soon be on a par. M. Boullay has analyzed the *viola odorata,* and found it to contain an active alkaline bitter and acrid principle, similar to the emetine of ipecacuanha. This he denominates the *violine.* Orfila has proved it to possess highly poisonous qualities. This principle resides equally in the root, leaves, flowers, and seeds, united with a malic instead of a gallic acid.

---

9. *Injection of Tartar Emetic into the Veins.* Dr. Meplain of Doujon, was the hardy experimenter, on this occasion. The subject was a young woman who had suffered from her infancy from worms, but in other respects was healthy. For several days previously to the report, she had had irregular and voracious appetite, and yet disgust at the sight or smell of food. Her nights were disturbed by frightful dreams, and she had frequent inclination to vomit. After a fatiguing journey she was seized in the evening with fever, intense headache, eructations, wandering pains in the limbs, cramps in the legs, delirium, grinding of the teeth. Next day, hysterical symptoms, convulsions, jactitation. In the second night, loss of consciousness, rigidity of the limbs. When Dr. M. arrived, he found the patient completely immobile—eyelids raised—eyes fixed—pupils contracted—head bent backwards—jaws locked—limbs tetanically rigid—pulse scarcely perceptible—skin cold—abdomen soft—urine white.

Dr. M. attributed these symptoms to the presence of worms in the stomach, or their attempts to ascend the œsophagus. He therefore endeavoured, by various means, to dislodge them by vomiting, without success. He next determined on the injection of an emetic into the veins. By means of a hydrocele syringe, he threw into the median vein six ounces of whey, holding in solution four grains of tartar emetic. The patient exhibited no sign of sensibility during the injection. In about twenty minutes, the eyes and lower lip began to move, and soon afterwards vomiting came on, and brought away eight lumbrici rolled up in a ball, all living. There was now a striking amendment of the patient's condition. The rigidity of the limbs ceased—the pulse became stronger. She again vomited, threw off two lumbrici of a larger size, after which she began to articulate, and soon recovered, having vomited a third time, and brought away several more lumbrici, and much bile.—*Journ. Comp. des Sciences Med. Fev.*

10. *Cases of Masked Intermittents.* By M. Bourquet, Surgeon in Chief to the Hospitals of Beziers.*

*Case* 1. M. Bourquet was summoned to Fau—, in the mountains, to see a male child, nealy 8 years old, with the following symptoms: Immediately at sun-rise he appeared to die away, and revived again at sun-set. M. B. wished to see these phenomena in person, and went to him at six o'clock in the morning, (it was winter) awoke him, and chatted with him until about sun-rise, when the little invalid, hitherto lively, lay down and soon appeared quite dead. No sensible respiration, no pulse, no beating of the heart, no feeling even when pricked. The body grew colder considerably for at least two hours, after which the cold diminished, without entirely ceasing; the only sign of life was a convulsive twitching of the right upper eye-lid. One of his arms was forcibly raised, it remained so; a leg, the same. His limbs were like soft wax which takes any form that is given to it. Thus he continued, without swallowing any thing, until the sun sank beneath the horizon, when he gradually recovered his senses without any sign of having been ill.

He had had two similar attacks previously, treated unsuccessfully with stimulants, rubefacients, &c. Bark in combination with sublimed zinc was ordered; during the night, this was taken to the amount of an ounce; no paroxysm appeared the next day, nor has any since.

*Case* 2. Mademoiselle J. T——, of Beziers, felt every day, precisely at 2 o'clock in the afternoon, a kind of epigastric cramp, which, after half an hour, induced so forced and loud a laugh that her mother thought her mad. This immoderate laughter continued eight hours, and was terminated by a sweating stage, when tranquillity re-appeared. The friends were not a little surprised to see these "laughing fits," as they called them, disappear under the use of bark, after baths, blood-lettings, and debilitating drinks had been employed in vain.

*Case* 3. Miss Fi— An— of C——, experienced, for two days, a chorea so violent, that she leaped continually, during more than two hours, half a foot high; striking herself; struggling in every direction; with incessant hiccough and contortions, until at length, worn out with fatigue, she sank upon a sofa, where a most profound slumber terminated the paroxysm. Her ordinary medical attendant ordered the application of leeches, which procured no amendment. M. Bourquet, considering the disease as similar to that of the former cases, employed the bark: the success was complete.

* Gazette de Santé. No xvii. Juin.

*Case* 4. Miss V. F——, of Beziers, felt, exactly every Thursday, dreadful colicky pains, with inflation of the lower belly; pulse hard; respiration laborious; convulsive motions perceptible. By turns, diluents, evacuants, and leeches, were essayed: nothing could ward off the attack. The intermittent character of the disease clearly called for bark: This was administered on the eve of the day when the paroxysm should come on; it did not appear and the patient was perfectly cured.

*Case* 5. M. Mignard, of Sauvian-les-Beziers, was attacked with febrile delirium, and soon an anthrax appeared on the thumb of the right hand. An opening was made at the end of the thumb, and the digital artery, being aneurismal, gave out, by a jet synchronous with the pulse, some quantity of blood. Compression stopped the hæmorrhage: it reappeared; again it was stopped, again it appeared; and M. Bouquet was called in either to tie the digital artery, or to amputate the thumb, if the case required it. This was on the day of the hæmorrhage, and it was remarkable that the discourse of the patient was vague and confused. It struck M. B. that this periodical bleeding was but the symptom of some nervous paroxysm, which was renewed every second day. The pulse was hard and sharp, and in the thumb affected, pulsations extremely violent were felt: the radial artery seemed to participate in the dilatation. The bare finger was applied, after having compressed the artery at the wrist, and the blood stopped by dossils sprinkled with agaric, a bandage and a tourniquet in readiness upon the radial. The next day, Wednesday, the patient was easy; on Thursday, the bleeding reappeared. On Friday, the following boluses were given: powdered bark 1 oz. extract of bark 1 dr. salt of bark ½ dr. made up with syrup of bark into 8 portions, one to be taken every four hours. The patient took them on Friday. *Saturday*, no hæmorrhage, no delirium, extraordinary heat. The thumb was examined and the escar removed, but not a drop of blood made its appearance. The cure was complete.

*Periodicity* should be carefully watched in all anomalous diseases; for the treatment requires, in general, to be reversed, when the intermittent nature of a complaint is ascertained. We have known much suffering and some danger incurred, in consequence of mistaking periodical paroxysms for occasional exacerbations of febrile diseases.

---

11. *Of Madeira, its Visitors, its Climate, and its Diseases.* Dr. Heineker of Funchal, Madeira, has published some observations upon these important subjects, and we regret extremely that the playful puerile way in which he has discussed them, induced us to look with an eye of severity upon his observations, which certainly contain many useful, and we doubt not well founded opinions upon the climate of Madeira, and more particularly upon the transference of patients, labouring under phthisis, or threatened with its attacks, from our own shores to that place. The careless apathy with which patients are frequently condemned to transportation, to separation from their friends, and to considerable expence, in a state of suffering which no change of climate can remedy, has frequently excited our regret, and we should be infinitely indebted to any man for a *serious* statement of the particular species and stages of pulmonic disease, which are likely to be relieved by the residence at Madeira. The subject is too grave to admit of trifling, and we wish Dr. Heineker had clothed his observations in a more sedate and serious garb. He very truly, however, states, that physicians too often advise, and pulmonics undertake, a voyage to Madeira, under erroneous impressions, and unfavourable circumstances; he concludes,

That a person with the *slightest inclination* towards consumption, can not seek a change of climate too early.

That a residence of a few winter months will avail but little.

That years, or at all events, winters, are necessary towards recovery or security.

That climate, unassisted by care and prudence, will be insufficient.

That a suppression of symptoms, for a time, is not to be trusted to—"*lateat scintilla.*"

That *incipient* is a frequent misnomer for *confirmed.*

That those in the more advanced stages of disease, are not to expect a *cure*, but only a *palliative*, and that, too, under the strictest precautions, and as long only as they breathe a bland and temperate air.

So many books have been published upon the climate, &c. of Madeira, that it is not necessary to refer to the very scanty remarks of Dr. H. upon this part of the subject.

For his concise statement of the prevailing diseases of Madeira, we must refer to the original paper, in the Repository of July.

---

12. *Sulphate of Quinine—Masked Intermittent.* We do not recollect seeing any case where this new remedy was taken, to any extent at least, by a medical man, and where, of course, the physiological effects of the medicine could be appreciated by personal feeling. The following concise statement may not, perhaps, be entirely unworthy of the reader's attention, on several accounts. We shall give it in the words of the patient himself, as noted in the intervals of his attacks, and we can vouch for the correctness of the statement.

"On Friday night (25th of June, 1824) I returned home late, the night being raw and damp. I had scarcely got into bed, when I was seized with a rigor, and a peculiarly painful sensation in every muscle of the body. This state continued several hours, and was not succeeded by any re-action; but, on the contrary, a chilliness, accompanied by the muscular pain, remained through the night. Next day, (*Saturday* 26*th*) kept in bed—pulse 80—skin cool—tongue furred—the muscles of the eye-balls so painful, that I dared scarcely to look around. *Sunday*, 27*th*—being much solicited, went out in the carriage, to see some patients, in a very uncomfortable state. The muscular soreness very great—no appetite at all—no thirst—no head-ache—nor any febrile symptom, except furred tongue, and some quickness of pulse. In the evening, a slight rigor, and such an increase of the muscular soreness, that I went to bed, and spent a wretched night, having horrible dreams, whenever I fell asleep. *Monday*, *28th.*—A good deal better to-day, and sat up; but the muscular soreness continued, and my intellect was clouded. The dismal dreams, and half-waking phantoms, continued to distress me greatly now, and throughout the whole of this week, gradually increasing in intensity. Nothing but aperients and diaphoretics had hitherto been taken. *Tuesday*, *29th.* Got up a while in the forenoon, but was harassed with indescribable languor, dejection of mind, and irritability of temper. At four, p. m. the rigor came on, and lasted about half an hour, succeeded by great heat of skin, pulse 110, thirst, and the most dreadful chaos of horrible images passing through my mind. In the evening, bled to syncope, and leeches applied to the temples and forehead. Spent a wretched night, feeling cold, yet covered with warm perspiration. *Wednesday*, *30th.* Somewhat better—no rigor—no fever—but the same dreadful depression of spirits, languor, and terrific images, when I dozed, or even closed my eyes. *Thursday*, *July 1st.* All the forenoon, felt an anticipation of an attack, and an increased perturbation of mind. Was seen by several physicians, who agreed that there was nothing serious in the complaint, as my pulse was only 86, skin cool—tongue moist, though furred—and no pain or uneasiness, except in my muscles. *At three o'clock*, *p. m.* the enemy made his approach with a severe rigor, of three-quarters of an hour's duration, succeeded by the most tremendous reaction, which I had ever felt—or, I think, seen. The heat suddenly got up, and the pulse rose to 136, every artery vibrating with intensity. But it was the intellectual suffering which absorbed all my attention. Though broad awake, and perfectly sensible, a rapid succession of the most terrific images perpetually presented themselves to my mind, while the most perfect conviction obtained that my last day was come. I became thoroughly convinced, from the dreadful state of my intellectual system, that the brain and its membranes were inflamed, even to disorganization and effusion. In more than one of my dreadful waking dreams, I conceived that I was lying on my face, in a dissecting room, while two anatomists were opening my head and spine, and descanting on the mass of disease which the parts presented! The horrors of that evening can never be forgotten. The images now, and indeed all along, were invariably of a sepulchral hue—tombs, skeletons, putrid bodies, and fearful spectres, were ever the prominent figures of the agonizing drama which passed before me, or in which I thought myself engaged. This dreadful hot stage changed into a profuse perspiration, which lasted the greater part of the night, with very little mitigation of suffering, and only transient dozes of sleep, or rather of terrifying dreams. My pulse, even next morning, was 110, though the heat and perspiration had ceased. For two or three days past, I had been suspecting that my disorder was assuming the tertian type, and was now convinced of it. But, as my pulse kept high this day (*2nd July*) my physicians wished to see whether the paroxysm should again return on Saturday, before they ventured on bark. Friday passed in the usual state of languor, depression of spirits, and sleeping and waking dreams of death and all his attributes. *Saturday, 3d July.* After a wretched night, I felt the usual precursor—an anticipation of the attack. As the clock struck two, the cold stage set in, and lasted three-quarters of an hour. Then came the reaction, similar to that of Thursday, but not accompanied with quite so much mental distress, or waking delirium, being now, as were all my medical friends, convinced that the disease was completely tertian. The hot and sweating stages, however, continued with great intensity, till midnight, when the perspiration began to abate. At this hour I commenced the sulphate of quinine, at first, in doses of two grains every four hours—but gradually increased, so that I took twenty grains before the period of accession, on Monday. No paroxysm, however, recurred that day, nor afterwards—but seven grains more of the sulphate were taken in the next thirty-six hours. The debility now felt was excessive—and a few grains of calomel, which had been taken more than a week previously produced ptyalism that lasted ten days.

"I have, more than once, remarked the despondency, and the train of sepulchral images, that haunted my imagination, not only on the days of the attacks, but during the imperfect remissions which intervened. After the second dose of the sulphate of quinine, these changed into a character diametrically opposite. Every image that now floated before the mind, whether waking or sleeping, was of the joyous and pleasing cast, nor could I, when I tried from curiosity, conjure up a single sombre spectre, of all the countless multitudes that before occupied the whole of my mental view. This I consider to be a very curious phenomenon, and a very remarkable illustration of the connexion and dependence subsisting between conditions of body and operations of mind. The only other physiological effect of the quinine, which I shall notice, is its influence on the vascular system. For several days after the medicine had been discontinued, the pulse kept strong and full, at from 100 to 108, without the accompaniment of any one other attribute of fever. The skin was cool—the tongue moist—no thirst, with all this strong tone of the vascular system. It could not have been the effects of the mercury, for when the mercurial influence became far stronger, the pulse fell to 80, and remained there during convalescence. From this unequivocal power which the quinine possesses, of increasing the tone of the heart and arteries, without apparently disturbing the other functions or systems, I apprehend we shall find, in this preparation, a remedy for many states and conditions of the body, to which the common forms of bark must ever be inapplicable, in consequence of the bulk of the medicine, and the disturbance which it produces in the stomach, and other digestive organs. The sulphate, even when taken in doses of three grains, never occasioned the slightest inconvenience to the stomach. On the contrary, I generally felt a pleasant warm sensation there, for an hour or two after taking the medicine. I hope these observations may prove useful, in leading to the exhibition of this powerful tonic, in diseases for which bark has not been given at all—or not with advantage."*

---

13. *Belladonna employed as a Preventive of Scarlatina.* There is no kind of knowledge which confers more honor upon the medical art, than that by which we are enabled to prevent the occurrence of disease. Every attempt that is made to achieve so noble, so philanthropic an object, should be deliberately and philosophically examined. By many practitioners, in Germany, belladonna is supposed to possess the singular power of preventing scarlet fever. It had been observed by Dr. Hahnemann, that very small doses of this drug produced symptoms analogous to those of scarlatina, and he was led to the hope that it might prove an antidote to the disease. Upon this highly important subject, Professor Koreff has addressed a letter to M. Laennec; the opinion, he maintains, is supported by the observation of sixteen years.† "Observation clearly proves," he says, "that the belladonna, taken for some time, either in powder or in extract, produces, especially in infants, a redness of the skin, which is sometimes transient, at others more durable. Dryness of the mouth, with a sensation of heat in the throat. dilatation of the pupil, anxiety; occasionally swelling of the submaxillary glands: symptoms having a great resemblance to those which accompany the eruption of scarlatina.

"The effect of the belladonna has also this, in common with scarlatina, that neither of them produces the redness of the skin invariably, whilst the symptoms about the throat are always present. It was not till I had received the authority of the celebrated Soemmering, who informed me that he obtained the most satisfactory results with it, when the disease raged epidemically, that I determined to employ it.

"This malady, accompanied by the most unfavourable symptoms, and having entirely changed its usual character, was, at that time, producing ravages almost as fatal as the contagious typhus. I then, for the first time, had to protect from this dreadful contagion almost all those who took the belladonna with a little perseverance, and of these there were many thousands.

"Since that time, I never lost sight of the discovery, which becomes the more valuable as the scarlatina has increased during the last thirty years, both in violence and extent, in many countries, and I have always found the same effects in different climates, and in epidemics of opposite characters. Many other physicians have equally confirmed the preventive powers of this plant, and the German Journals are daily filled with proofs of a benefit, which, with respect to some countries, equals that of vaccination. In France, the capital and provinces of which appear less subject to these fatal epidemics, than Germany, Switzerland, &c. less attention has been given to this discovery, and it

---

* I forgot to mention the quinine produced considerable torpor of the bowels, which required much aperient medicine.

† Sur l'Emploi de la Balladone contre la Contagion de la Scarlatine—Bulletin des Scienees Medicales, Avril, 1824.

has been rejected—it must be said, too lightly, and without any sufficient examination, as may be seen in the article, belladonna, in the Dict. des Sc. Med. I only remember a single observation upon this important subject, by Dr. Meglin, who gives an account of a trial which he gave to this preservative, during an epidemic of scarlatina at Colmar, and which confirms all the assertions of the German physicians. The absence of present danger is, perhaps, the cause of this indifference towards a discovery which, important in itself, might also be fruitful in results applicable to other diseases. At present, however, I shall confine myself to an account of the results which have been ascertained by repeated observations, and by a great number of individuals placed in very different circumstances, without incurring the reproach of having proceeded in a manner not sufficiently rigorous. The powder mixed with sugar, or the extract carefully made from the juice of the recent plant, are employed after the following formulæ. Extract of belladonna, three grains, dissolved in an ounce of cinnamon water. Powder or root of belladonna, two grains mixed with ten drachms of white sugar, divided into sixty doses. From half a dose to a whole one is given to a child, from six months to two years old, four times a day. To children from three to six years old, from a dose to one and a half. To those from six to nine, two to two and a half. To those from ten to twelve, three to four and a half. Of the solution, a drop is given for every year of the child's age, once a day, and fasting. Observation has shewn, that when the epidemic is very fatal, or the intercourse with the patients very frequent and intimate, it is prudent to increase the dose a little. It has not yet been possible to determine, in a satisfactory manner, the length of time which is necessary to eradicate, by this remedy, the susceptibility of the contagion. Every thing leads us to believe that the remedy, if used during a time too short to ward off the contagion, moderates very much the malignity of the disease. We know, for certain, that the remedy does not permanently overcome the disposition to scarlatina; and it is necessary to resume its use on every recurrence of an epidemic. We have always observed that the most intimate communication with the sick does not produce the disease, provided the medicine has been employed eight or nine times previous to being exposed to the contagion, and continued up to the period of desquamation. A circumstance very important to nurses. It appears more certain, to begin with rather strong doses, in order to guard against the first impression of the contagion, and to diminish the quantity after a few days. No sensible effect has been observed to follow the continued use of this small quantity of belladonna. Up to the present time, neither season nor locality, nor any other circumstance, has appeared to diminish the preservative effect of this plant."

To us, the above facts do not appear very extraordinary. We know that, with few exceptions, two diseases will not go on in the same body at the same time. What is the effect of medicine but a disease? If then, from the use of a particular remedy, local and constitutional symptoms were produced, similar to those of scarlatina, we should certainly have imagined, without being in possession of the above statement of the German practitioner, that during the continuance of those symptoms, scarlatina would be very unlikely to take place. There are many well-known analogous facts, which will immediately occur to the minds of our readers, and upon which we need not dwell, to prove the infrequency with which two different morbid actions proceed simultaneously in the same patient. In this country, it is true, scarlatina is generally a mild disease. We may, however, be visited with severe and fatal epidemics, which would render necessary a trial of the plan proposed above.

